# Seroprevalence of *Angiostrongylus cantonensis* in suspected human angiostrongyliasis cases and clinical manifestations in a tertiary care hospital in Thailand: a retrospective 18-year longitudinal study

**DOI:** 10.1016/j.fawpar.2025.e00290

**Published:** 2025-09-15

**Authors:** Lakkhana Sadaow, Thidarat K. Prasongdee, Phuangphaka Sadee, Sureeporn Naonongwai, Patcharaporn Boonroumkaew, Rutchanee Rodpai, Oranuch Sanpool, Amnat Kitkhuandee, Verajit Chotmongkol, Kittisak Sawanyawisuth, Pewpan M. Intapan, Wanchai Maleewong

**Affiliations:** a Department of Parasitology, Faculty of Medicine, Khon Kaen University, Khon Kaen 40002. Thailand; b Mekong Health Science Research Institute, Khon Kaen University, Khon Kaen 40002, Thailand; c Clinical Immunology Unit, Srinagarind Hospital, Faculty of Medicine, Khon Kaen University, Khon Kaen, Thailand; d Department of Medical Technology, Faculty of Allied Health Sciences, Nakhonratchasima College, Nakhon Ratchasima 30000, Thailand; e Department of Surgery, Faculty of Medicine, Khon Kaen University, Khon Kaen 40002, Thailand; f Department of Medicine, Faculty of Medicine, Khon Kaen University, Khon Kaen 40002, Thailand

**Keywords:** Angiostrongyliasis, Clinical manifestations, Seroprevalence, Eosinophilic meningoencephalitis, Foodborne parasite

## Abstract

Human angiostrongyliasis, a foodborne parasitosis caused by *Angiostrongylus cantonensis*, is primarily characterized by eosinophilic meningitis (EOM), meningoencephalitis or myelitis. This study reports the seroprevalence of *A. cantonensis* among suspected cases of angiostrongyliasis and describes the clinical manifestations in patients with positive serological tests at a Thai tertiary care hospital over an 18-year period (2006–2023). Among 768 patients suspected of eosinophilic meningitis related to *A. cantonensis* infection, 353 (46 %) tested positive for *A. cantonensis* IgG antibodies. Most seropositive patients were from the northeast region of Thailand (308/353; 87.2 %). Among the seropositive patients, 13 % reported a history of consuming uncooked freshwater snails or other paratenic hosts. Headache was reported in 23.5 % of the patients, with 31.3 % (*n* = 26) experiencing acute severe headache. Fever and neck stiffness were present in 6.8 % and 6.5 % of the patients, respectively, while nausea and vomiting were observed in 7.9 %. In a subset of 56 seropositive patients who underwent lumbar puncture, cerebrospinal fluid (CSF) examination was clear in 49 (87.5 %), cloudy in six (10.7 %), and xanthochromic in one case (1.8 %). The highest recorded CSF opening pressure was 600 mmHg, with a median pressure of 210 mmHg. The average CSF white blood cell count was 200 cells/mm^3^ (range: 0–2250 cells/mm^3^), with eosinophils constituting an average of 35 % (range: 1–90 %). These findings may be useful for clinicians in endemic regions as supportive information for clinical diagnosis.

## Highlights

• 18-year longitudinal seroprevalence study on human angiostrongyliasis is documented.
• Characterized by eosinophilic meningitis, meningoencephalitis, or myelitis
• 768 suspected eosinophilic meningitis cases were related to *A. cantonensis* infection.
• A total of 353 cases tested positive for IgG antibodies against *A. cantonensis*
• History of hazardous food consumption and clinical manifestations are revealed

## 1 Introduction

Angiostrongyliasis, mainly caused by *Angiostrongylus cantonensis*, was first discovered in Guangzhou, China in 1935 (Chen, 1935). The disease is primarily characterized by eosinophilic meningitis (EOM), meningoencephalitis or myelitis (Wang et al., 2008). At least 3161 cases of the disease have been recorded globally (Puthiyakunnon and Chen, 2015). Thailand and China remain the two main endemic countries (Wang et al., 2012; Ansdell and Wattanagoon, 2018; Federspiel et al., 2020). Moreover, angiostrongyliasis has been recognized as an emerging disease among travelers, with a review reporting at least 84 cases involving travelers to and from endemic areas across 15 countries (Meesing et al., 2023). Recently, a retrospective analytical study reported epidemiological data on eosinophilic meningitis from government hospitals submitted to Thailand's Bureau of Epidemiology, part of the Ministry of Public Health's Department of Disease Control, between 2014 and 2019 (Khamsai et al., 2022). This study documented a total of 1083 cases, with an average annual incidence of 180.5 cases (0.27 per 100,000 population) (Khamsai et al., 2022). The northeast zone of Thailand exhibited the highest rate at 0.89 per 100,000 population (Khamsai et al., 2022; Graeff-Teixeira et al., 2023).

Humans are accidental hosts of this zoonotic pathogen and can become infected by ingesting larvae found in raw or undercooked snails, other paratenic hosts, contaminated water or vegetables. Once ingested, the third-stage larvae migrate via the bloodstream to the central nervous system, where they cause EOM, often accompanied by encephalitis and myelitis (meningo-encephalo-myelitis) (Cross, 1978). The most common symptom in angiostrongyliasis patients is acute headache without accompanying neurological deficits (Khamsai et al., 2020). In addition, parasite larvae or immature adults can invade the eyes, producing various ocular symptoms, including optic neuritis (Diao et al., 2011; Feng et al., 2013; Boonroumkaew et al., 2020). In Thailand, the consumption of raw *Pila* snails is the primary transmission route for EOM (Barratt et al., 2016). This traditional habit, known locally as “koi hoi,” has resulted in the highest number of reported EOM cases in the country (Eamsobhana et al., 2010). Although *A. cantonensis* infection leads to EOM, clinical meningitis is observed in only 15.2 % of cases (Khamsai et al., 2020). This may lead to misdiagnosis or underdiagnosis. Recently, a diagnostic criterion was proposed that defines proven angiostrongyliasis cases as those in which an *Angiostrongylus* worm is found in the cerebrospinal fluid (CSF) or eyes, or when a positive DNA test is obtained (Graeff-Teixeira et al., 2023). Unfortunately, proven cases are rarely found in clinical practice or reported in literature. Generally, presumptive diagnosis is based on clinical presentation, a history of mollusk ingestion, eosinophilic pleocytosis in the CSF, and advanced neuroimaging, while serological tests (Barratt et al., 2016) and DNA detection methods (Sears et al., 2021) provide supportive evidence of EOM-associated angiostrongyliasis. In Thailand, a clinical diagnosis is commonly made when eosinophils constitute 10 % or more of the total CSF white blood cells (Khamsai et al., 2020). However, studies on the seroprevalence and clinical manifestations of EOM-associated angiostrongyliasis remain limited. This study, therefore, aimed to report the seroprevalence of *A. cantonensis* in suspected angiostrongyliasis cases and the clinical manifestations in those with positive serological tests in a tertiary hospital over an 18-year period.

## 2 Materials and methods

This hospital database study was conducted at Khon Kaen University Hospital in northeastern Thailand. Researchers retrospectively searched the hospital database for suspected angiostrongyliasis cases and included patients who had undergone serological testing for *A. cantonensis* during an 18-year period from 2006 and 2023. Suspected cases were defined as patients presenting with symptoms consistent with *A. cantonensis* infection such as headache, fever, and neck stiffness or those considered at risk due to the consumption of raw or undercooked freshwater snails or paratenic hosts within the past six months (Khamsai et al., 2020).

**Fig. 1:**
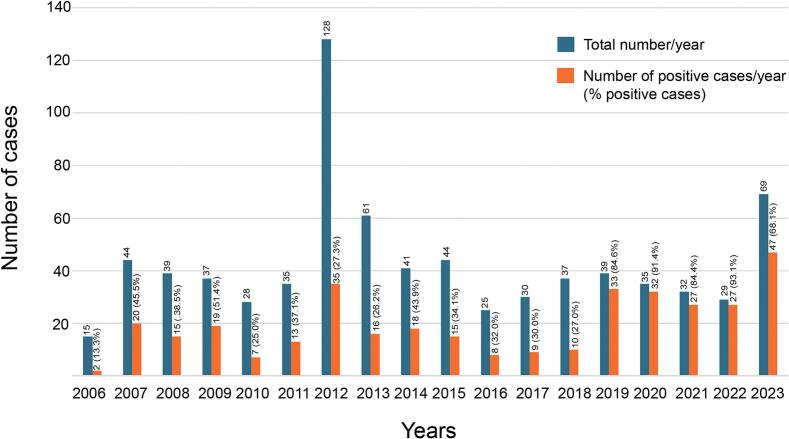
Distribution of patients seropositive for *Angiostrongylus cantonensis* (353, 46%) in a series of suspected angiostrongyliasis cases (n =768) by year between 2006 and 2023.

Data were collected on the number of suspected angiostrongyliasis cases, which were tested for the presence of *A. cantonensis* specific IgG antibody using an in-house immunoblotting assay targeting the 29 kDa antigenic band of *A. cantonensis* young adult female worm somatic extract (Sawanyawisuth et al., 2011), or a recombinant *A. cantonensis* galectin-2 based immunochromatographic test (ICT) kit (Somboonpatarakun et al., 2020). The sensitivity and specificity of the assays were reported for immunoblotting as 56.6 % and 99.4 % (Sawanyawisuth et al., 2011), respectively, and for the ICT as 87.0 % and 96.5 % (Somboonpatarakun et al., 2020), respectively. Cases testing positive for *A. cantonensis* IgG antibody in either test were classified as seropositive. The data were categorized by year, as well as by geographical distribution (Fig. 1, Table 1). Thailand comprises 76 provinces and the capital city, Bangkok, which are grouped into six regions based on geographical features: north, northeast, central, east, west, and south. The numbers of suspected and seropositive cases for each region and province were recorded accordingly. The clinical features of seropositive angiostrongyliasis cases were evaluated based on baseline characteristics, clinical presentation, and laboratory tests. Laboratory assessments included blood tests such as the complete blood count, serum albumin, serum globulin, and serum total protein and CSF analyses, which included color, opening/closing pressure, total and differential white blood cell counts, protein, glucose, and the CSF/plasma glucose ratio.

**Table 1:** Distribution of patients seropositive for *A. cantonensis* in a series of suspected angiostrongyliasis cases (*n* = 768) by province of Thailand.

Provinces	Total tested	Positive	% positive
**North**	**1**	**0**	**0**
Chiang Mai	1	0	0
**Northeast**	**669**	**308**	**46.0**
Khon Kaen	245	124	50.6
Maha Sarakham	58	25	43.1
Kalasin	66	30	45.4
Chaiyaphum	41	19	46.3
Loei	34	14	41.2
Roi Et	33	10	30.3
Sakhon Nakhon	26	12	46.1
Udon Thani	25	11	44.0
Nong Khai	21	11	52.4
Nong Bua Lam Phu	21	11	52.4
Surin	16	8	50.0
Nakhon Phanom	15	5	33.3
Buri Ram	14	4	28.6
Bueng Kan	10	2	20.0
Ubon Ratchathani	10	4	40.0
Yasothon	9	4	44.4
Si Sa Ket	9	6	66.7
Nakhon Ratchasima	7	2	28.7
Mukdahan	7	5	71.4
Amnat Charoen	2	1	50.0
**Central**	**6**	**3**	**50.0**
Bangkok	2	0	0
Phetchabun	2	2	100
Phitsanulok	1	1	100
Nakhon Nayok	1	0	0
**East**	**1**	**0**	**0**
Chanthaburi	1	0	0
**South**	**1**	**1**	**100**
Phuket	1	1	100
**Loa PDR**	**8**	**4**	**50.0**
**Not available**	**82**	**37**	**45.1**
Total	768	353	46.0

The data were analyzed using descriptive statistics and are presented either as a count (percentage) or as the median with its range. Statistical analyses were performed with STATA software, version 18.1 (College Station, Texas).

## 3 Results

During the 18-year period (Fig. 1), there were 768 suspected cases of eosinophilic meningitis associated with *A. cantonensis* infection. Among these, 353 cases (46 %) tested positive for *A. cantonensis* IgG antibody. On average, there were 42.7 suspected cases per year (SD 24.6) and 19.6 seropositive cases per year (SD 11.7). The highest number of suspected cases was 128 in 2012, while the peak number of seropositive cases was 47 in 2023.

In terms of geographical distribution (Table 1), the highest number of suspected cases (669 out of 768; 87.1 %) was found in the northeast region, with Khon Kaen province reporting the highest count within that region (245 cases, representing 36.6 % of the region's suspected cases). Additionally, there were eight suspected cases from the Lao People's Democratic Republic and six from Thailand's central region. Similarly, most seropositive cases were found in the northeast region (308 out of 353; 87.2 %), with Khon Kaen province having the highest number (124 out of 308; 40.3 %). Among the patients investigated, no seropositive case came from Chiang Mai, Bangkok, Nakhon Nayok, or Chanthaburi.

Clinical characteristics of seropositive cases were shown in Table 2. The median patient age was 50 years, ranging from as young as one year old to as old as 86 years old. Males accounted for 61.2 % of the cases. Only 13.0 % of the seropositive individuals reported a history of consuming raw freshwater snails or other paratenic hosts. Headache was the most common symptom, experienced by 83 patients (23.5 %). Headache was described as acute, with a proportion of acute severe headache of 31.3 % (*n* = 26). Additionally, fever and neck stiffness were observed in 24 (6.8 %) and 23 (6.5 %) patients respectively. Nausea/vomiting was the second most common symptom, affecting 28 patients (7.9 %).

**Table 2:** Clinical characteristics of patients seropositive for *A. cantonensis* (*n* = 353) in a series of suspected angiostrongyliasis cases.

Factors	Results
Age, years	50 (1–86)
Male sex	216 (61.2 %)
History of consuming uncooked snails or other paratenic hosts	46 (13.0 %)
**Symptoms & signs**	
Headache	83 (23.5 %)
Acute headache	57 (16.1 %)
Acute severe headache	26 (7.4 %)
Fever	24 (6.8 %)
Neck stiffness	23 (6.5 %)
Cranial nerve palsy	4 (1.1 %)
Nausea/ vomiting	28 (7.9 %)
Hemiplegia/ hemiparesis	8 (2.3 %)
Motor weakness	30 (8.5 %)
**CSF characteristics**	
Color	*N* = 56
Clear	49 (87.5 %)
Cloudy	6 (10.7 %)
Xanthochromic	1 (1.8 %)
Opening pressure, mmHg	210 (80–600)
Closing pressure, mmHg	160 (80–600)
White blood cells, cells/mm^3^	200 (0–2250)
Eosinophils, %	35 (1–90)
Neutrophils, %	7 (1–92)
Lymphocytes, %	45 (1−100)
Monocytes, %	7 (1–76)
Protein, mg/dL	77 (0.2–2181)
Glucose, mg/dL	52 (5–137)
CSF glucose/ plasma glucose, %	58.12 (31.1–154.0)
**Other laboratory tests**	
Hemoglobin, g/dL	12.8 (6.4–17.1)
Hematocrit, %	39.1 (18.5–51.1)
White blood cell, x10^3^ cells/mm^3^	8.5 (1.3–39.3)
Eosinophils, %	6.9 (0–84.8)
Neutrophils, %	58.6 (4–96.7)
Lymphocytes, %	22.5 (2.0–68.5)
Monocytes, %	5.7 (0.3–25.6)
Serum albumin, g/dL	4.1 (2.1–5.1)
Serum globulin, g/dL	3.1 (1.9–5.9)
Serum total protein, g/dL	7.3 (3.1–11.8)

There were 56 seropositive patients who underwent lumbar puncture (Table 2). Most CSF examinations showed a clear appearance (49 cases; 87.5 %), six cases (10.7 %) had cloudy CSF, and one case (1.8 %) displayed xanthochromia. The highest CSF opening pressure was 600 mmHg with a median of 210 mmHg. The average CSF white blood cell count was 200 cells/mm^3^, ranging widely between 0 and 2250 cells/mm^3^.The average percentage of CSF eosinophils was 35 %, with a low value of 1 %. The median CSF protein level was slightly elevated at 77 mg/dL, reaching as high as 2181 mg/dL. The median white blood cell count was 8500 cells/mm^3^, with a median eosinophil percentage of 6.9 % and a maximum of 84.8 %. Serum albumin and globulin had median values of 4.1 g/dL and 3.1 g/dL, respectively.

## 4 Discussion

This longitudinal study showed that the number of suspected angiostrongyliasis cases was quite steady over the 18-year study period, with a peak in 2012 which may indicate an outbreak. Similarly, seroprevalence rates of angiostrongyliasis during the study period were also steady, with an average of 46 %. Since the study was conducted at a tertiary care hospital, the actual number of suspected and seropositive cases of angiostrongyliasis may be higher than reported since not all patients experiencing similar signs and symptoms may be expected to reach tertiary care. The results of the present study are comparable to those of a previous national database study conducted over a six-year period, where the annual number of reported eosinophilic meningitis cases in Thailand ranged from 144 to 204 patients (Khamsai et al., 2022). Most cases were from the northeastern region (90.1 %) (Khamsai et al.,2022), which is consistent with our findings, where 87.1 % of patients also originated from the northeast. One key difference between the two studies is that the national database study included patients with eosinophilic meningitis regardless of serological confirmation, whereas the current study only reports on seropositive cases.

Regarding the clinical manifestations of angiostrongyliasis in patients with a positive serological test, most features were consistent with previous reports (Khamsai et al., 2020; Wang et al., 2008, Wang et al., 2012). The most common symptom was acute headache, which was severe in one third of the cases. Unlike in acute bacterial meningitis, the clinical sign of neck stiffness, which is suggestive of meningitis, was found in only 6.5 % of patients, which may lead to underdiagnosis or misdiagnosis. Notably, our finding is in sharp contrast with a previous study which has shown meningeal signs in approximately 40–50 % of cases of eosinophilic meningitis caused by *A. cantonensis* (Khamsai et al., 2020).

Motor weakness and hemiparesis were not frequent among our cases (exhibited by 8.5% and 2.3%). These manifestations are generally uncommon in angiostrongyliasis; for example, a study from Vietnam reported that only 3.2 % of patients with eosinophilic meningitis and positive PCR exhibited hemiplegia (McBride et al., 2017). Therefore, in the diffrential diagnosis of patients presenting with hemiparesis it is also important to consider gnathostomiasis (Senthong et al., 2013).

A cloudy CSF, resembling coconut juice, is typical of eosinophilic meningitis caused by *A. cantonensis* (Wang et al., 2012), although this pathognomonic feature is uncommon. In this study, 10.7 % of CSF samples exhibited a cloudy appearance. The CSF opening pressure reached as high as 600 mmHg in approximately 40 % of cases, with the highest CSF white blood cell count recorded at 2250 cells, similar to a previous study (Sawanyawisuth and Chotmongkol, 2013). Notably, the CSF white blood cell count was normal (fewer than 5 cells/mm^3^) in 11 patients, less than 10 % eosinophils in the CSF were found in five, while more than 70 % neutrophils in the CSF were found in four patients. These findings indicate that some seropositive angiostrongyliasis cases may not meet the CSF criteria for eosinophilic meningitis (Sawanyawisuth and Chotmongkol, 2013). Since the CSF criteria can be inconclusive, a history of eating raw freshwater snails or other paratenic hosts is crucial for diagnosing angiostrongyliasis (Graeff-Teixeira et al., 2023). Blood eosinophilia also supports the diagnosis (Graeff-Teixeira et al., 2023; Sawanyawisuth and Chotmongkol, 2013), however, this study found a median percentage of blood eosinophils of 6.9 %. An earlier study suggested that a blood eosinophil count exceeding 798 cells, in combination with a history of consuming raw or undercooked freshwater snails, may be indicative of eosinophilic meningitis (Sawanyawisuth et al., 2010).

This study has several limitations. First, the results of the serological methods employed should be interpreted cautiously. Potential cross-reactions may occur in some cases of mixed infections, particularly with fascioliasis when tested with immunoblotting (Maleewong et al., 2001), as well as with trichinosis, ascariasis, hookworm infections, opisthorchiasis, and sparganosis (Somboonpatarakun et al., 2020). Background antibodies from previous infections with *Angiostrongylus* spp. may also interfere with the results. Second, some clinical data were missing due to incomplete medical records, particularly concerning the history of exposure to transmission agents, clinical presentation, and laboratory tests. Note that details of CSF were reported in only 56 patients as most patients refused a lumbar puncture. However, a presumable diagnosis of angiostrongyliasis based on serological tests may be justified as these tests have good diagnostic properties. Although gnathostomiasis and baylisascariasis are other possible causes of eosinophilic meningitis, there are major differences in the clinical presentation and/or epidemiology of these two diseases and angiostrongyliasis. Gnathostomiasis commonly presents with motor weakness or migratory swelling (Senthong et al., 2013; Khamsai et al., 2021), while baylisascariasis has never been reported in Thailand and raccoons are not commonly found in Thailand (Alonso Aguirre et al., 2019). Finally, clinical outcomes were not evaluated.

## 5 Conclusions

The seroprevalence of *A. cantonensis* in suspected angiostrongyliasis cases remained relatively steady over an 18-year period. The clinical manifestations in patients with positive serological tests were generally consistent with previous reports, although some differences were noted in CSF white blood cell counts, as well as in eosinophil levels both in the CSF and blood. Developing awareness to the range of variable blood and CSF eosinophil counts is important for clinicians dealing with suspected meningitis in areas endemic for angiostrongyliasis. Given that numerous cases have been reported over nearly two centuries, the implementation of robust public health control measures remains essential to reducing the morbidity and mortality associated with this helminthic disease.

## CRediT authorship contribution statement

**Lakkhana Sadaow:** Writing – review & editing, Writing – original draft, Software, Methodology, Investigation, Formal analysis, Data curation, Conceptualization. **Thidarat K. Prasongdee:** Resources, Methodology, Investigation, Formal analysis, Data curation, Conceptualization. **Phuangphaka Sadee:** Resources, Methodology, Investigation, Formal analysis, Data curation, Conceptualization. **Sureeporn Naonongwai:** Resources, Methodology, Investigation, Formal analysis, Data curation, Conceptualization. **Patcharaporn Boonroumkaew:** Resources, Methodology, Investigation, Formal analysis, Data curation, Conceptualization. **Rutchanee Rodpai:** Resources, Methodology, Investigation, Formal analysis, Data curation, Conceptualization. **Oranuch Sanpool:** Methodology, Investigation, Formal analysis, Data curation, Conceptualization. **Amnat Kitkhuandee:** Resources, Conceptualization. **Verajit Chotmongkol:** Resources, Conceptualization. **Kittisak Sawanyawisuth:** Writing – review & editing, Writing – original draft, Supervision, Software, Resources, Conceptualization. **Pewpan M. Intapan:** Writing – review & editing, Writing – original draft, Supervision, Resources, Funding acquisition, Conceptualization. **Wanchai Maleewong:** Writing – review & editing, Writing – original draft, Supervision, Funding acquisition, Conceptualization.

## Declaration of competing interest

The authors declare that they have no known competing financial interests or personal relationships that could have appeared to influence the work reported in this paper.

The ethical guidelines and principles of the Declaration of Helsinki guided all study procedures. The Ethics Committee for Human Research at Khon Kaen University approved this study (HE621019). All data were fully anonymized, and the Human Ethics Committee waived the requirement for informed consent. During the preparation of this work the authors used [ChatGPT-4.0] for assistance in checking and refining the English language in this manuscript including minor translations. The author entirely generated the content, ideas, and findings presented in the manuscript without AI assistance. After using this tool/service, the authors reviewed and edited the content as needed and take full responsibility for the content of the publication.
